# Treatment interruption in hypertensive patients during the COVID‐19 pandemic: An interrupted time series analysis using prescription data in Okayama, Japan

**DOI:** 10.1002/jgf2.678

**Published:** 2024-02-21

**Authors:** Naoko Nakamura, Toshiharu Mitsuhashi, Naomi Matsumoto, Shunsaku Hayase, Takashi Yorifuji

**Affiliations:** ^1^ Department of Epidemiology Okayama University Graduate School of Medicine, Dentistry and Pharmaceutical Sciences Okayama Japan; ^2^ Center for Innovative Clinical Medicine Okayama University Hospital Okayama Japan; ^3^ Academic Affairs Division Okayama University Okayama Japan

**Keywords:** antihypertensive agents, COVID‐19, health behavior, interrupted time series analysis, prescription drugs, treatment interruption

## Abstract

**Background:**

The COVID‐19 pandemic has impacted healthcare behaviors, leading to fewer pediatric visits in Japan and potentially fewer visits by adult patients. However, existing Japanese studies on treatment interruptions have generally relied on questionnaire‐based methods. In this study, we assessed the impact of the pandemic on antihypertensive treatment interruption using real‐world prescription data.

**Methods:**

We conducted an interrupted time series analysis using the National Health Insurance Database in Okayama Prefecture, Japan. Participants included individuals aged 40–69 years with at least one antihypertensive prescription between 2018 and 2020. Treatment interruption was defined as a 3‐month or longer gap in prescriptions after medication depletion. We used segmented Poisson regression with models unadjusted and adjusted for seasonality and over‐dispersion to assess monthly treatment interruptions before and after Japan's April 2020 emergency.

**Results:**

During the study period, 23.0% of 55,431 participants experienced treatment interruptions. Cyclical fluctuations in interruptions were observed. The crude analysis indicated a 1.2‐fold increase in treatment interruptions following the pandemic; however, the adjusted models showed no significant changes. Even among higher‐risk groups, such as women, younger adults, and those with shorter prescriptions, no significant alterations were observed.

**Conclusion:**

We found no significant impact of the COVID‐19 pandemic on antihypertensive treatment interruption in Okayama Prefecture. The less severe outbreak in the area or increased use of telemedicine and extended prescriptions may have contributed to treatment continuity. Further research is needed using a more stable and comprehensive database, broader regional data, and detailed prescription records to validate and extend our findings.

## Graphical abstract

We conducted an interrupted time series analysis using the National Health Insurance Database in Okayama Prefecture, Japan, to assess the impact of the COVID‐19 pandemic on antihypertensive treatment interruptions. The study found no significant increase in treatment interruptions during the pandemic.

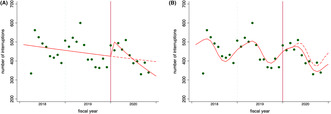

## 1 INTRODUCTION

The global spread of COVID‐19 has fundamentally reshaped people's everyday lives, including medical consultation behaviors. An analysis of insurance claim databases in the United States revealed increased prescription interruptions for various chronic diseases among adults during the COVID‐19 pandemic.^1^ Treatment interruptions are particularly problematic in hypertension, where consistent treatment is crucial for controlling blood pressure and preventing cardiovascular events.^2^ Such interruptions can also lead to excessive healthcare costs.^2^ This concern underpins our focus on antihypertensive treatment interruption within this study.

During the initial wave of the pandemic in Japan, a notable decrease in patient numbers was observed, especially in pediatrics^3^; this has been substantiated by a prescription database.^4^ In a similar trend, adults undergoing treatment for chronic diseases were also presumed to avoid medical consultations for fear of contracting COVID‐19. Although Japanese studies^5^, ^6^, ^7^ have demonstrated an increase in treatment interruptions among adult patients during the COVID‐19 pandemic, these studies were all based on questionnaires. Self‐reported assessments of adherence to antihypertensive medications are generally inaccurate due to patients' tendencies to overestimate their adherence.^8^ On the contrary, prescription databases provide objective information without the self‐reporting biases inherent in questionnaire‐based research. However, to the best of our knowledge, no studies have verified the issue of treatment interruption for hypertension using prescription databases.

In the present study, we used actual prescription practices to verify whether the COVID‐19 pandemic affected treatment interruption among patients with hypertension. Furthermore, through stratified analysis based on variables such as sex and age, we aimed to identify any groups that were particularly vulnerable to treatment interruption.

## 2 METHODS

### 2.1 Design, setting, and participants

We conducted a quasi‐experimental study using interrupted time series (ITSs) analysis to evaluate the impact of the COVID‐19 pandemic on the number of hypertension treatment interruptions among patients in Japan. To investigate this in actual clinical practice, we used the National Health Insurance (NHI) database, also known as the Kokuho Database (KDB), which is owned by the Okayama National Health Insurance Organization. The KDB encompasses monthly insurance claim data, allowing us to track a patient's medical information irrespective of changes in their healthcare institution, as long as they remain enrolled in the NHI.

The source population in this study comprised NHI beneficiaries residing in Okayama Prefecture. Okayama is located in the western part of Japan and had a population of approximately 1.9 million in 2020.^9^ Approximately 20.1% of the population (380,000 individuals) were enrolled in the NHI during fiscal year 2020.^10^ NHI beneficiaries primarily include self‐employed individuals, farmers, part‐time workers not covered by the Employee's Health Insurance, retirees, and their dependents.

In this study, we included individuals aged between 40 and 69 years as of the end of fiscal year 2018 (i.e., March 2018). This age group was selected because the prevalence of hypertension increases significantly from age 40 onward^11^ and including individuals above the upper age limit could result in demographic instability due to the transition of those aged 75 and over to the Late‐stage Elderly Medical Care System during the study period. In addition, participants were required to have been prescribed any of the 59 specified antihypertensive drugs at least once during the study period from fiscal year 2018 to 2020. These drugs were identified according to their generic names from the following drug classes that are recommended for initial treatment in the Guidelines for the Management of Hypertension 2019^12^: calcium antagonists; angiotensin II receptor blockers; angiotensin‐converting enzyme inhibitors; thiazide diuretics; and combinations of these drugs.

Participants who died or were hospitalized during the study period were excluded. In addition, we excluded those who were diagnosed with pregnancy‐induced hypertension with fewer than 10 months of antihypertensive drug prescriptions; by definition, pregnancy‐induced hypertension resolves in fewer than 10 months after onset, inherently causing treatment interruption. The diagnosis of pregnancy‐induced hypertension was determined as International Classification of Diseases, Tenth Revision codes O11 and O13 through O16, except O142 (Hemolysis, Elevated Liver enzymes, and Low Platelets syndrome), thereby including gestational hypertension, preeclampsia, and eclampsia.^13^ We also excluded individuals with fewer than seven prescription days in total during the study period, considering the unlikelihood of sustained treatment.

### 2.2 Intervention and outcome

In this study, the onset of the COVID‐19 pandemic was defined as April 1, 2020, the month when the Japanese government issued its first declaration of a state of emergency in response to the rapid domestic spread of infection.^14^ This declaration generated fears about infection and anxiety about strains on the healthcare system, leading to widespread behavioral changes throughout Japan, even in areas with less severe outbreaks.^15^ We hypothesized that these changes affected treatment interruptions, including treatment for hypertension.

We defined treatment interruption as a situation in which there was no prescription for three or more consecutive months following the month when the prescribed amount of medication was depleted. This definition was based on prior studies^16^, ^17^ that have used a lack of consecutive prescriptions for more than 90 days as an indicator of significant interruption of antihypertensive medication. To identify the occurrence of treatment interruptions, for each individual, we determined the months with prescriptions, the months without prescriptions but covered by previous prescriptions, and the months with neither cover nor prescriptions. Because the KDB does not provide the exact prescription date, we assumed that prescriptions were provided at the end of the month, defining the coverage periods as follows: prescriptions for 1–30 days covered the following 1 month; those for 31–60 days covered the following 2 months; those for 61–90 days covered the following 3 months, and prescriptions for 91 days or more covered the following 4 months. If the definition of interruption was met, we considered the interruption to have occurred in the month when the coverage expired. After identifying the months of interruptions for each individual, we counted the number of interruptions in each month and reconstructed this as time series data.

### 2.3 Statistical analysis

We used ITS analysis to evaluate the impact of treatment interruption for hypertension owing to the COVID‐19 pandemic, an intervention that affected the entire target population. As a crucial method for assessing the effect of population‐level interventions at specific points in time, ITS provides visual insights and statistical evidence regarding changes in outcomes before and after the intervention, achieved through longitudinal plots of outcomes and corresponding regression analysis.^18^ In this study, we modeled the monthly count of treatment interruptions using segmented Poisson regression. According to the existing literature on the COVID‐19 pandemic and prescriptions,^19^, ^20^ we used an impact model that assumed immediate level and slope changes following the intervention. That is, we included interaction terms between time (in months since the onset of the intervention) and intervention in the segmented regression model.

In time series data, the outcomes often have a seasonal pattern, leading to autocorrelation, which violates the regression model's assumption that error terms are independent and that there should be no correlation between observations.^21^ Additionally, the observed variance may exceed that assumed by the Poisson distribution, a phenomenon known as over‐dispersion.^18^ To address these issues, we used Fourier terms for seasonality and added a chi‐squared scale parameter to the model, allowing the variance to be proportional rather than equal to the mean. Any remaining autocorrelation was assessed by plotting the autocorrelation and partial autocorrelation functions of the residuals across multiple lags.

We plotted the monthly number of treatment interruptions on a scatter graph, overlaying it with the modeled predictive trend and a counterfactual scenario based on the pre‐pandemic trend. This visualization helped with assessing whether any changes occurred related to the pandemic. We also estimated the coefficients to derive the level and slope change in the number of treatment interruptions before and after the pandemic. Specifically, the change in level was presented as ratios, whereas the change in slope was expressed indirectly by showing the multiplicative trends for each period. These findings were presented along with their 95% confidence intervals.

To investigate whether specific subgroups were more susceptible to the impact of treatment interruptions, we conducted a stratified analysis based on sex, age group (40s, 50s, and 60s), and the presence or absence of 90‐day prescriptions defined as prescriptions for 90 days or more. Furthermore, to assess the robustness of our primary analysis, we conducted a sensitivity analysis by shifting the intervention time points^22^ to the first day of April, July, and October in 2019, when no actual interventions occurred. All analyses were conducted using Stata SE version 17 (StataCorp LLC, College Station, TX, USA).

## 3 RESULTS

### 3.1 Participant characteristics

Of 78,565 NHI beneficiaries aged 40–69 years with antihypertension prescriptions between fiscal years 2018 and 2020, we included 55,431 participants in this study after exclusions for the following reasons: 1259 deaths, 21,668 hospitalizations, two cases of pregnancy‐induced hypertension, and 205 patients with temporary prescriptions. The characteristics of participants are summarized in Table 1. Participants were approximately equally divided by sex, and most were in their 60s. The median of the individual average days supplied per prescription was 33.3; patients with 90‐day prescriptions only comprised slightly more than 10%. Treatment interruption was experienced by 23.0% (*n* = 12,760) of the total population. Although multiple interruptions could occur per person given the definition, 90.8% (*n* = 11,580) had only a single interruption. Of these, 9358 individuals, or 73.3% of all with interrupted prescriptions, did not resume treatment within the study period.

**TABLE 1:** Characteristics of 55,431 National Health Insurance beneficiaries, aged 40–69 years, who participated in this hypertension treatment interruption study in Okayama, Japan (2018–2020).

Sex, *n* (%)	
Male	28,325 (51.1)
Female	27,106 (48.9)
Age, median (IQR), year	65 (60–68)
Age group, *n* (%) (years)	
40–49	3691 (6.7)
50–59	8631 (15.6)
60–69	43,109 (77.8)
90‐day prescription, *n* (%)	6311 (11.4)
Average prescription days, median (IQR), day	33.3 (30.0–54.6)
Diagnosis of essential hypertension (ICD‐10 code: I10), *n* (%)	54,994 (99.2)
Treatment interruption, *n* (%)	12,760 (23.0)

Abbreviations: ICD‐10, International Classification of Diseases, Tenth Revision; IQR, interquartile range.

### 3.2 Description of prescriptions and interruptions

**FIGURE 1:**
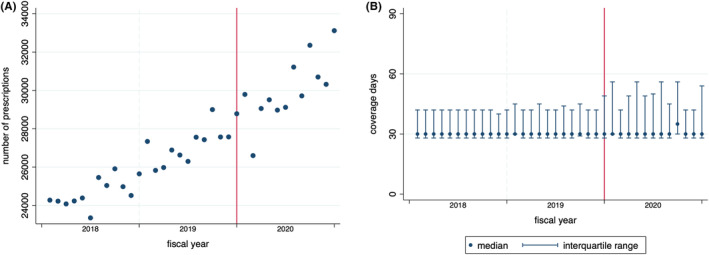
Monthly trends in (A) number of prescriptions and (B) days covered by prescriptions in Okayama, Japan.

**FIGURE 2:**
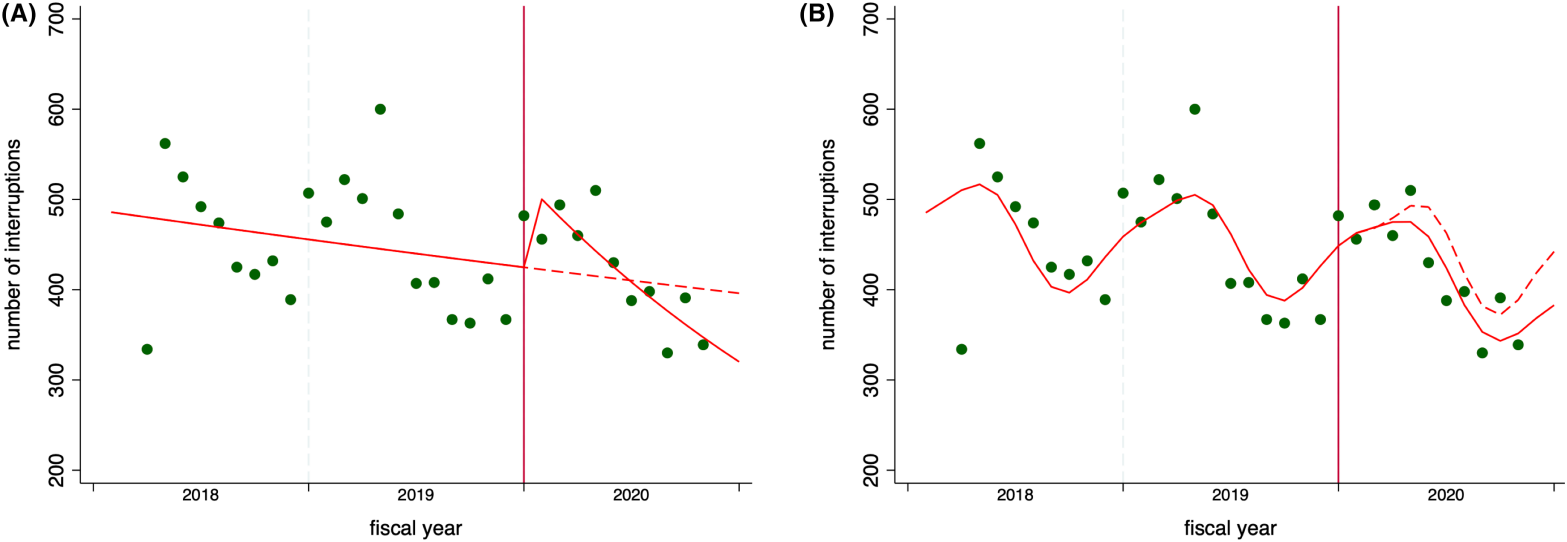
Monthly number of hypertension treatment interruptions in Okayama, Japan, with predicted trends from (A) crude and (B) seasonality‐adjusted models. Dots: observed interruptions. Solid line: predicted trend. Dashed line: counterfactual scenario.

According to Figure 1A, the monthly prescription count showed a general upward trend. The dots in Figure 2 suggest a yearly cyclical variation in the monthly number of treatment interruptions. In Table 2, we present the median numbers of monthly hypertension prescriptions and treatment interruptions before and after the COVID‐19 pandemic. It reveals the increase in the median prescription numbers and either a slightly decreased or stable median number of patients with treatment interruption after the pandemic across all strata.

**TABLE 2:** Descriptive analysis for the median number of monthly hypertension prescriptions and treatment interruptions before and after the COVID‐19 pandemic in Okayama, Japan.

	Pre‐pandemic	Post‐pandemic	Pre‐pandemic	Post‐pandemic
	Median interruptions (per month)	Median interruptions (per month)	Median prescriptions (per month)	Median prescriptions (per month)
Overall	453	414	25,872	29,754
Male	234	226	12,928	14,935
Female	205	189	12,952	14,820
Age 40–49 years	54	54	1205	1422
Age 50–59 years	103	104	3324	3812
Age 60–69 years	286	254	21,303	24,528
Without 90‐day prescription	426	370	23,884	27,599
With 90‐day prescription	33	38	1990	2067

The median number of prescription days remained roughly constant, but the interquartile range expanded slightly after the onset of the pandemic, as depicted in Figure 1B.

### 3.3 Main findings

Figure 2 shows the predicted lines based on the segmented Poisson regression model with counterfactual lines assuming no pandemic. Table 3 presents the results of the statistical analysis. In the crude analysis, the number of treatment interruptions increased by approximately 1.2 times immediately after the onset of the pandemic, and the decreasing trend in treatment interruptions accelerated slightly from an approximately 1% decrease per month during the pre‐pandemic period to an approximately 4% decrease per month after the pandemic. However, in the adjusted model, no significant changes were observed in either the level or trend. This finding was consistent across all strata (Table 3) and was confirmed in analyses by changing the intervention point (Table S1).

**TABLE 3:** Immediate and trend changes in monthly hypertension treatment interruptions before and after the COVID‐19 pandemic in Okayama, Japan: estimates from a segmented Poisson regression analysis.

	Crude			Adjusted^a^		
	Pandemic onset	Pre‐pandemic	Post‐pandemic	Pandemic onset	Pre‐pandemic	Post‐pandemic
	Level change ratio^b^ (95% CI)	Multiplicative trend^c^ (95% CI)		Level change ratio^b^ (95% CI)	Multiplicative trend^c^ (95% CI)	
Overall	1.23 (1.14 to 1.32)	0.99 (0.99 to 1.00)	0.96 (0.95 to 0.97)	1.01 (0.81 to 1.26)	1.00 (0.99 to 1.01)	0.99 (0.95 to 1.03)
Male	1.17 (1.06 to 1.30)	0.99 (0.99 to 1.00)	0.97 (0.95 to 0.99)	1.02 (0.82 to 1.27)	1.00 (0.99 to 1.00)	0.99 (0.95 to 1.03)
Female	1.28 (1.16 to 1.43)	1.00 (0.99 to 1.00)	0.95 (0.93 to 0.97)	1.00 (0.77 to 1.30)	1.00 (0.99 to 1.01)	0.98 (0.93 to 1.03)
Age 40–49 years	0.89 (0.72 to 1.10)	1.01 (1.00 to 1.02)	1.01 (0.97 to 1.05)	0.84 (0.57 to 1.25)	1.01 (0.99 to 1.02)	1.01 (0.94 to 1.08)
Age 50–59 years	1.08 (0.93 to 1.25)	1.00 (1.00 to 1.01)	0.98 (0.95 to 1.01)	0.91 (0.72 to 1.15)	1.01 (1.00 to 1.02)	1.00 (0.95 to 1.04)
Age 60–69 years	1.37 (1.25 to 1.50)	0.99 (0.98 to 0.99)	0.94 (0.93 to 0.96)	1.09 (0.86 to 1.39)	0.99 (0.98 to 1.00)	0.98 (0.93 to 1.02)
Without 90‐day prescription	1.26 (1.17 to 1.36)	0.99 (0.99 to 0.99)	0.96 (0.94 to 0.97)	1.04 (0.83 to 1.31)	0.99 (0.99 to 1.00)	0.98 (0.94 to 1.02)
With 90‐day prescription	0.90 (0.70 to 1.15)	1.03 (1.02 to 1.04)	0.99 (0.95 to 1.04)	0.69 (0.37 to 1.28)	1.04 (1.02 to 1.07)	1.02 (0.91 to 1.14)

Abbreviation: CI, confidence interval.

^a^ The model includes a scaling adjustment to address over‐dispersion and a Fourier term to account for seasonality.

^b^ Pandemic onset‐level change ratio represents the ratio of the change in the number of individuals with treatment interruption from before to after the onset of the COVID‐19 pandemic.

^c^ Pre‐pandemic and post‐pandemic multiplicative trends each represent the monthly fold‐change in the number of individuals with treatment interruption during their respective periods.

Regarding the autocorrelation function and partial autocorrelation function of residuals in the adjusted model (Figure S1), neither exceeded the 95% confidence interval, indicating that no significant autocorrelation remained after adjustment.

## 4 DISCUSSION

In our study using ITS analysis, we found no significant impact on treatment interruptions among hypertensive patients owing to the COVID‐19 pandemic in Japan, even among groups considered to be at higher risk for treatment interruptions such as women,^16^, ^23^ younger individuals,^16^, ^24^ and those without 90‐day prescriptions.^17^ This finding differs from the results of existing studies.^1^, ^25^

Our findings suggest that the impact of the pandemic on treatment interruptions was limited within the population targeted in our study. Akashi et al.^6^ reported a positive correlation between the level of COVID‐19 spread in each prefecture and chronic disease treatment interruptions. In April 2020, Okayama reported only 0.05 new COVID‐19 infections per 100,000 people, markedly lower than 0.89 in Tokyo and a national average of 0.33.^26^ Although this low incidence may appear to have influenced the lack of increased number of treatment interruptions in Okayama, we cannot confirm this because a regional comparative analysis was not conducted in our study. Nevertheless, it is important to consider the context of Okayama's relatively minor outbreak in the interpretation of our findings.

An alternative interpretation could be that the reduction in outpatient visits did not increase treatment interruptions. As reported by Takakubo et al.,^5^ 37.8% of respondents said that they completed fewer outpatient visits during the COVID‐19 pandemic, but approximately one‐sixth of that number said they had depleted their regular medication. This observed gap resembles the disparity between our expectation of an increase in hypertension treatment interruptions during the pandemic and the actual findings of our study, suggesting that patients may have continued treatment without clinic visits. A possible method for this could have been to extend the number of prescription days. Previous research using prescription data showed an increase in the prescription count and extended prescription days during the initial phase of the pandemic.^1^, ^4^ This indicated a possible effort by patients and healthcare providers to minimize clinic visits during periods with rapid infection spread by extending prescriptions in advance. In our study, such temporary cessation of clinic visits following extended prescriptions can be differentiated from treatment interruptions, which are defined as depletion of the prescribed medication. Thus, our findings suggested that even if more patients avoided outpatient visits, this did not necessarily lead to an increase in treatment interruptions in an essential sense. As shown in Figure 1B, although no change was observed in the median number of prescription days, expansion of the interquartile range after the onset of the pandemic implies that there was a slight increase in the number of individuals receiving extended prescriptions.

Another possible explanation for the lack of an increase in treatment interruptions was the growth in telemedicine. Ishikawa et al. found that the proportion of telemedicine use increased approximately threefold immediately following the first COVID‐19 emergency declaration in Japan, with most telemedicine provided via telephone and for prescription purposes.^27^ In our study, we focused on monthly prescriptions, regardless of whether they were in‐person or via telephone; therefore, the exact proportion is unclear. However, it is possible that telephone prescriptions contributed to the continuation of treatment during the pandemic.

### 4.1 Strengths

A key feature of the present study was our objective evaluation, which was based on actual prescriptions extracted from the KDB. Furthermore, the detailed outcome definition, focusing on prescription coverage, allowed us to assess actual treatment interruptions as distinguished from cessation of clinic visits.

In our study, we effectively utilized the strengths of ITS. In single‐group ITS analysis, the pre‐intervention trend projected onto the post‐intervention period serves as a counterfactual scenario, which allows for a robust evaluation even when it is challenging to establish a comparable control group.^28^ Moreover, this approach enabled us to disregard confounders assumed to be constant or to change slowly within a population.^28^ This is particularly advantageous when using the KDB, which lacks extensive information on factors such as smoking habits and socioeconomic status.

### 4.2 Limitations

The KDB encompasses data on NHI beneficiaries. Consequently, if individuals switch to Employees' Health Insurance or relocate out of the prefecture, their data are discontinued in the KDB from that point. Using these data, it becomes challenging to distinguish whether a prescription interruption for hypertensive patients was stemming from withdrawal from the NHI or an actual treatment interruption. Among patients we counted as having treatment interruptions, some might have left the NHI. In particular, as seen in Figure 2, the increase in treatment interruptions at the onset of a fiscal year could be influenced by heightened NHI withdrawals, which are common owing to employment changes or relocations during fiscal transitions. Given that our intervention point, April 2020, is aligned with the start of the Japanese fiscal year, the increase in treatment interruptions at this time would have paralleled the previous cyclical trends, and adjusting for this could have masked the true impact of the pandemic. However, even when we conducted evaluations using multiple placebo intervention points, including April in other years, the results remained consistent, supporting the finding of no impact of the COVID‐19 pandemic on hypertension treatment interruptions.

In contrast to data discontinuation when beneficiaries leave the NHI, the KDB begins capturing data upon their enrollment. The upward trend in prescription numbers, as observed in Figure 1A, could result not only from the increasing need for hypertension treatment owing to aging but also from an influx of new NHI enrollees, who have withdrawn from the Employees' Health Insurance upon reaching retirement age at age 60 or 65 years. Thus, it should be noted that this trend might differ from the real‐world trend in terms of the number of individuals receiving antihypertensive drugs. Although the population receiving prescriptions (who are therefore at risk for treatment interruption) increased toward the end of the study period, there was a slight decrease in interruptions during the post‐pandemic period (Table 2). This suggests that the risk of treatment interruption following the onset of the COVID‐19 pandemic may be lower than that indicated in our study.

## 5 CONCLUSION

In our study, we did not identify a significant impact owing to the COVID‐19 pandemic in Japan on treatment interruption among hypertensive patients in Okayama Prefecture. Potential reasons for this include measures to prevent treatment interruptions via extended prescriptions and remote consultations. Although it is important to carefully consider the limitations inherent to our database, our findings contribute to the understanding of patient behavior during a global public health crisis such as a pandemic, which will be helpful in maintaining chronic disease management. Further investigations, particularly across a broader region and using more detailed prescription records and a more stable database of population outflows and inflows, are necessary to validate and extend our findings.

## FUNDING INFORMATION

(This work was partly) supported by the commissioned project budget for the ‘FY2021 National Health Insurance Health‐Up Support Project’, Okayama Prefecture.

## CONFLICT OF INTEREST STATEMENT

This work was partly supported by funding from the commissioned project budget “FY2021 National Health Insurance Health‐Up Support Project,” provided by Okayama Prefecture. The sponsors were not involved in the study design; the collection, analysis, and interpretation of data; the writing of the report; or the decision to submit the article for publication.

## ETHICS APPROVAL STATEMENT

This study was conducted in accordance with the Declaration of Helsinki. The study was approved by the Institutional Review Board of Okayama University Graduate School of Medicine, Dentistry, and Pharmaceutical Sciences (number 2201‐012).

## Supporting information


Data S1

## Data Availability

The datasets generated and analyzed in this study are not publicly available owing to institutional and data protection regulations.
