# Supplementary material for: Treatment interruption in hypertensive patients during the COVID‐19 pandemic: An interrupted time series analysis using prescription data in Okayama, Japan
Source: J Gen Fam Med. 2024 Feb 21;25(2):102–9. doi: 10.1002/jgf2.678 (PMC10927915; doi:10.1002/jgf2.678)
Supplement: Supplementary file 1 — Data S1 [file JGF2-25-102-s001.pdf]

[Supporting information]

Supplementary Table 1. Evaluation of monthly hypertension treatment interruptions around placebo intervention points.

	Crude			Adjusted[c]		
Intervention point	Intervention onset level change ratio[a] (95% CI)	Pre-intervention multiplicative trend[b]	Post-intervention (95% CI)	Intervention onset level change ratio[a] (95% CI)	Pre-intervention multiplicative trend[b]	Post-intervention (95% CI)
April 2020 (Actual)	1.23 (1.14 to 1.32)	0.99 (0.99 to 1.00)	0.96 (0.95 to 0.97)	1.01 (0.81 to 1.26)	1.00 (0.99 to 1.01)	0.99 (0.95 to 1.03)
April 2019 (Placebo)	1.13 (1.06 to 1.21)	0.99 (0.98 to 1.00)	0.99 (0.97 to 1.01)	0.91 (0.75 to 1.10)	1.02 (1.00 to 1.06)	0.99 (0.94 to 1.05)
July 2019 (Placebo)	0.99 (0.93 to 1.06)	1.01 (1.00 to 1.01)	0.99 (0.97 to 1.00)	0.95 (0.80 to 1.12)	1.01 (0.99 to 1.03)	0.99 (0.96 to 1.03)
October 2019 (Placebo)	0.87 (0.81 to 0.93)	1.01 (1.00 to 1.01)	1.00 (0.98 to 1.01)	0.94 (0.80 to 1.11)	1.00 (0.99 to 1.01)	0.99 (0.97 to 1.02)

Abbreviation: CI, confidence interval.
[a]Intervention onset-level change ratio represents the ratio of the change in the number of individuals with treatment interruption from before to after onset of the intervention.
[b]Pre-intervention and Post-intervention multiplicative trends each represent the monthly fold-change in the number of individuals with treatment interruption during their respective periods.
[c]The model includes a scaling adjustment to address over-dispersion and a Fourier term to account for seasonality.

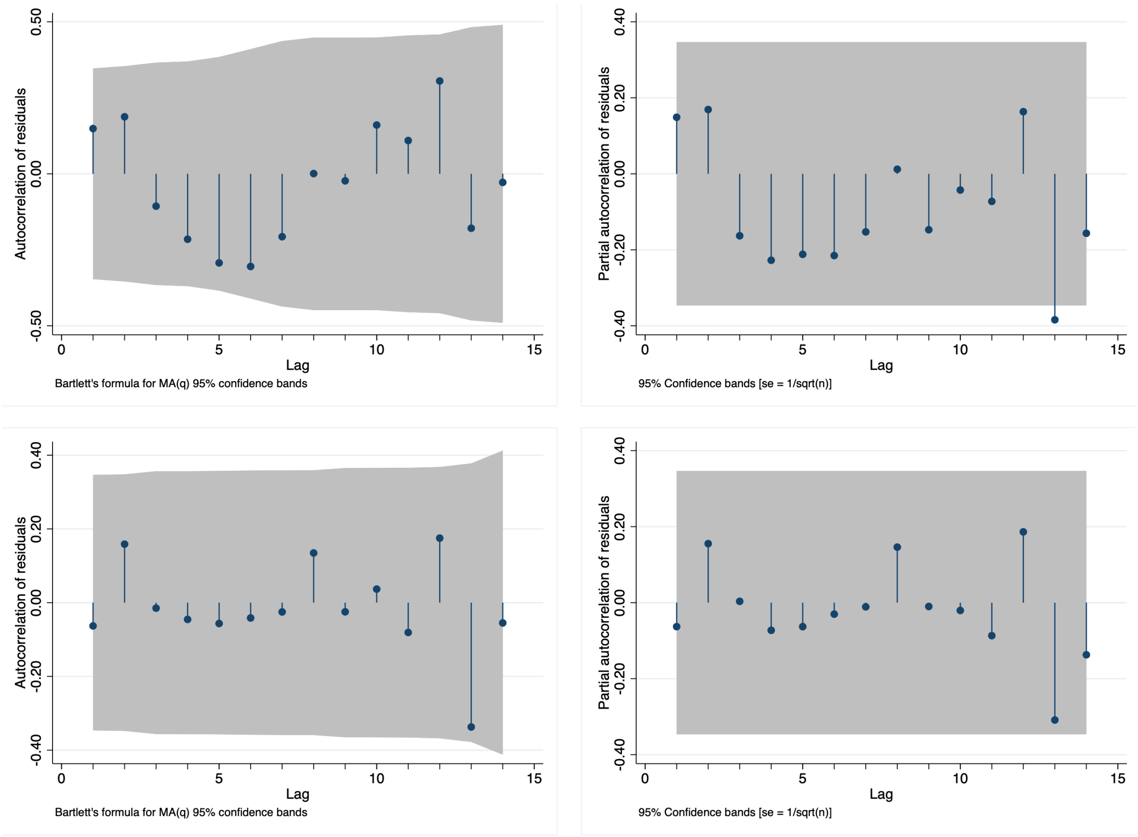

Supplementary Figure 1. Autocorrelation function (left) and partial autocorrelation function (right) of residuals. Upper: before seasonal adjustment. Lower: after seasonal adjustment.
